# Epidemiology of fractures in children and adolescents: Increased incidence over the past decade: a population-based study from northern Sweden

**DOI:** 10.3109/17453671003628780

**Published:** 2010-03-31

**Authors:** Erik M Hedström, Olle Svensson, Ulrica Bergström, Piotr Michno

**Affiliations:** Division of Surgery and Perioperative Science, Umeå University Hospital, UmeåSweden

## Abstract

**Background and purpose:**

Fractures are most common in youth and in the elderly, with differences in incidence over time and between regions. We present the fracture pattern in a population of youths **≤** 19 years of age, who were seen at Umeå University Hospital, Sweden.

**Material and methods:**

All injuries seen at the hospital have been recorded in a database since 1993. The data include variables such as age, sex, date, type of injury, mechanism of injury, and treatment. For the period 1993–2007, there were 10,203 injury events that had resulted in at least 1 fracture.

**Results:**

The incidence for the whole period was 201/10^4^ person years. The incidence increased by 13% during the period 1998–2007, when we were able to control for registration errors. The most common fracture site was the distal forearm. The most common type of injury mechanism was falling. The peak incidence occurred at 11–12 years in girls and at 13–14 years in boys, with a male-to-female incidence ratio of 1.5. We found variations in mechanisms and activities at injury with age, and over time.

**Interpretation:**

Fractures are caused by a combination of intrinsic and extrinsic factors that vary with age. We believe the increase in incidence is partly explained by changes in children's activity patterns over time. Further research may help to identify preventive measures to reduce the number of fractures, in particular those involving hospital care, surgical treatment, and—most importantly—long-term impairment.

## Introduction

Fractures are most common in youth and in old age when the skeleton is porous, with weak points at the physes and metaphyses, respectively. Around one-third of all children suffer at least one fracture before the age of 17 (Cooper et al. 2004), and fractures are the cause of 9% of all injuries in children that come to the attention of health services (Spady et al. 2004).

Previous studies have shown little variation in the distribution of fracture sites and mechanisms of injury; however, they have shown differences in incidence over time and between geographic regions (Table 1). Over the past few years, there has been concern about overweight and indolence in children. Is this reflected in the fracture pattern? We describe the incidence of fractures in a population of children and teenagers ≤ 19 years of age who were seen at Umeå University Hospital (UUH), Sweden. We also describe age- and sex-related differences in incidence, in mechanism of injury, and in fracture location.

**Table 1.** Overview of epidemiological studies describing children's fractures

First author	Age group	Study period	Location	Incidence per 10^4^	Most common fracture site	Most common mechanism of injury
Landin	0–16	1950–1979	Sweden	212	Distal forearm 23%	Falls
Cooper	0–17	1988–1998	Great Britain	133	Forearm 30%	N.A.
Kopjar	0–12	1992–1995	Norway	128	Distal radius 27%	Falls 71%
Tiderius	0–16	1993–1994	Sweden	193	Distal forearm 26%	Falls on ground level 40%
Lyons **^a^**	0–14	1996–1996	Scandinavia	156–178	Forearm 20%	Falls
Lyons **^b^**	0–14	1996–1996	Wales	361	Forearm 36%	Falls
Brudvik	0–15	1998–1998	Norway	245	Distal forearm 27%	N.A.
Rennie	0–15	2000–2000	Scotland	202	Distal forearm 33%	Falls < 1 m 37%
Our results	0–19	1993–2007	Sweden	201	Distal forearm 26%	Falls < 0.5 m 24%
**^a^** Lyons' results from districts in Sweden, Norway, and Finland.						
**^b^** Lyons' results from a Welsh district.						

## Material and methods

Umeå, Sweden, situated on the Gulf of Bothnia (64° N), has substantial seasonal variations in weather conditions. Snow and ice are common between November and April. The area is mainly urban and offers possibilities for a variety of sports and leisure activities. All children who sustain fractures in the municipality of Umeå and surrounding municipalities are seen at UUH, which serves as the regional hospital. During the period 1993–2007, when the data used here were collected, the average annual population aged 0–19 in the region was 34,239.

Injuries seen at the emergency department of the hospital were recorded in a database in accordance with EHLASS (the European Home and Leisure Accident Surveillance System). Data included variables such as age and sex, date, type and mechanism of injury, activity at injury, and treatment. Information was gathered through a questionnaire handed to patients or relatives upon arrival. Further information was gathered from reports by ambulance personnel, from the physician's notes on the case history, and from other possible sources—such as police reports. Non-residents were excluded. Residents injured elsewhere were registered if they were identified at follow-up visits. The database was cross-checked with the hospital's registry of inpatients, to avoid loss of injuries leading to admittance in the dataset. Starting from 1998, the registry of the emergency department's reception desk was also cross-checked to estimate the number of cases lost due to lack of registration among discharged patients.

From the database, we extracted 10,203 injury events in individuals aged 0–19 years that resulted in 10,327 fractures. Almost all fractures were confirmed radiographically; fractures of the nose and ribs were sometimes diagnosed by clinical examination only. We categorized the degree of trauma associated with each fracture event as slight, moderate, or severe in accordance with Landin's “severity of trauma” definitions (1983).

To validate fracture site recording, we reviewed the radiographs of 10% randomly selected cases from the years 2002–2007. These years were chosen because the films were digitized. For long bone fractures we used a definition of proximal, diaphyseal, and distal in accordance with AO principles, where the proximal and distal segments are defined by the broadest part of the physis (Slongo and Audigé 2007). Only 81% of the sample had been categorized correctly with respect to fracture site, and this led us to review all radiographs for the years 2006 and 2007 (1,520 cases). Thus, fracture site distribution is presented for these 2 years only.

When analyzing activity at injury, we divided the population into age groups with respect to enrollment in daycare and school and “milestones” in development.

### Definitions

Falls on the same plane were falls on ground level or from a height of < 0.5m. Falls between planes were defined as falls from a height of > 0.5 m. Downhill falls were defined as falls when moving on a downhill surface—on skis, a snowboard, or a sledge for example. Education-related fractures occurred on school grounds during classes or during intermissions.

### Statistics

We used SPSS version 14.0 for analysis of data noting incidence, fracture type, and mechanism of injury, including sex- and age-related differences. All incidence figures were calculated as incidence density and are presented per 10^4^ person years with 95% confidence intervals (CIs), if not stated otherwise. The population at risk used for each year and age was an approximated mid-year population, using data obtained from Statistics Sweden. For comparisons over time, we calculated age- and sex-adjusted, or age-adjusted and sex-specific incidence. We used Swedish national figures for the year 2000 as the standard population. To evaluate the significance of differences over time and between groups, we calculated incidence ratios with 95% confidence intervals.

## Results

**Figure 1.**
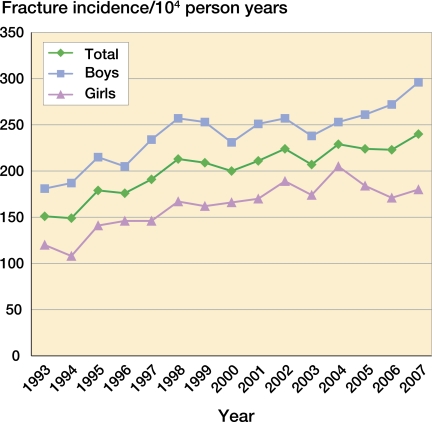
Age- and sex-adjusted incidence for both boys and girls and age-adjusted sex-specific incidence over time.

The incidence for the entire period was 201 (197–205) per 10^4^. The yearly incidence increased from 151 to 240, with an incidence ratio of 1.6 (CI: 1.4–1.8) (Figure 1 and Table 2). The incidence for children 0–16 years of age was 208 (204–212). The accumulated risk of sustaining a fracture before 17 years of age was 34%. The crude incidence of admittances due to fractures was 40 (38–42). The lowest incidence of admittance occurred in 1994 and 1997, 36 (29–42), and the highest incidence occurred in 2007, 51 (43–59).

**Table 2.** Number of fractures, population at risk, and age- and sex-adjusted incidence for each year

Year	Fractures	Population at risk	Incidence /10^4^ person years
1993	499	34,661	151
1994	497	34,961	149
1995	612	35,206	179
1996	604	35,275	176
1997	657	35,097	191
1998	728	34,776	213
1999	715	34,490	209
2000	685	34,136	200
2001	733	33,824	211
2002	775	33,686	224
2003	712	33,641	207
2004	790	33,641	229
2005	769	33,534	224
2006	763	33,386	223
2007	788	33,273	240

The lack of registration of injuries resulting in discharge for 1998–2007 was estimated to be 7%. There were variations over time in the percentage of cases lost due to lack of registration but the percentage was similar at the beginning and the end of the period.

**Figure 2.**
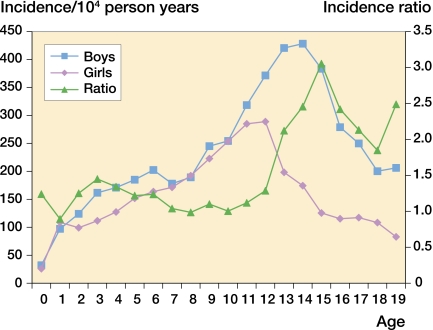
Sex-specific fracture incidence and incidence ratios for each year of age.

Boys accounted for 61% of all fracture events. The overall male-to-female incidence ratio was 1.5 (CI: 1.5–1.6). The highest incidence ratio between sexes occurred in the 15-year-olds: 3.1 (CI: 2.6–3.6). The peak incidence of fractures occurred at 11–12 years of age in girls and at 13–14 years of age in boys (Figure 2). There were seasonal variations with peaks in March, May-June, and August-September. The least number of fractures occurred in December.

### Fracture site

The most common fracture site was the distal forearm, with a peak in girls and boys at 11 and 14 years of age (Table 3). The sex-specific incidence was 74 (CI: 65–83) and 43 (CI: 36–50) for boys and girls, respectively, with an incidence ratio of 1.7 (CI: 1.4–2.1). The most common fracture site requiring admittance was the distal forearm (24%), followed by the tibial/fibular shaft (13%), and the forearm shaft (11%).

**Table 3.** Distribution of fracture sites, crude incidence, and percentage of all fractures, 2006–2007

Fracture site	Number of fractures	Incidence/10^5^ person years	Proportion (%)
Distal forearm	393	591	26
Clavicle	163	245	11
Fingers	149	224	10
Ankle	108	162	7
Toes	83	125	5
Metatarsals	79	119	5
Distal humerus	78	117	5
Metacarpals	78	117	5
Facial skeleton	51	77	3
Tibial/fibular shaft	47	71	3
Proximal forearm	46	69	3
Forearm shaft	46	69	3
Proximal humerus	38	57	3
Carpals	32	48	2
Proximal tibia/fibula	18	27	1
Ribs	17	26	1
Femoral shaft	15	23	1
Thoracolumbar spine	14	21	1
Distal femur	13	20	1
Tarsals	12	18	1
Humeral shaft	10	15	1
Skull	8	12	1
Other sites	22	33	1

### Mechanism

The most common type of injury mechanism was falls (Table 4). Fractures of the long bones were more often caused by falls whereas fractures of the axial skeleton, hand, and foot were more often caused by collisions, other blunt trauma, and traffic accidents. For example, 72% of fractures of the forearm shaft were caused by falls while 38% of skull fractures were caused by traffic accidents. Downhill falls accounted for 38% of tibial/fibular shaft fractures.

**Table 4.** Distribution of injury mechanisms

Injury mechanism	Events	Proportion (%)
Fall on the same plane	2,489	24
Collision with/struck by physical object, another person, or animal	2,407	24
Fall between planes	2,319	23
Traffic	1,227	12
Downhill fall	1,099	11
Crushed, cut or stuck	334	3
Other	328	3
Total	10,203	100

**Figure 3.**
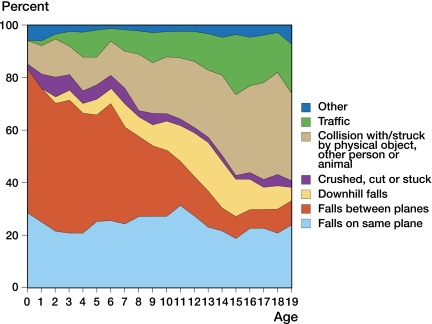
Distribution of injury mechanisms for each year of age.

Falls between planes were most common in the first years of life, with a marked decline after the age of 12. During the first year of life, falls between planes accounted for 54% of all fractures but only 6% in the 17-year-olds (Figure 3). The percentage of traffic-related fractures increased with age. Bicycles and mopeds accounted for 66% and 14% of traffic-related injuries, respectively. There was a seasonal variation in traffic-related injuries and downhill falls. The traffic-related injuries occurred for the most part in April to October, when the ground is clear of snow. Downhill falls reached their peak in late winter, February and March, when they were the cause of 30% and 42% of all fracture events.

The degree of trauma that caused fractures was categorized as slight (62%), moderate (30%), severe (5%), or unknown (3%). The trauma causing fractures that led to hospitalization was categorized as slight (50%), moderate (35%), severe (13%), or unknown (2%). Over time, the proportion of trauma leading to hospitalization that was categorized as slight decreased (55% to 41%) while the proportion of trauma categorized as moderate or severe increased (42% to 50%).

### Activity

Sport and play contributed to the most fracture events: 39% and 37%, respectively. Play dominated as the activity at injury during the first decade of life, whereas sport was the predominant activity in teenagers. There was a statistically significant increase in all activities over time in both boys and girls. No single activity was disproportionally responsible for the total increase in fractures.

**Figure 4.**
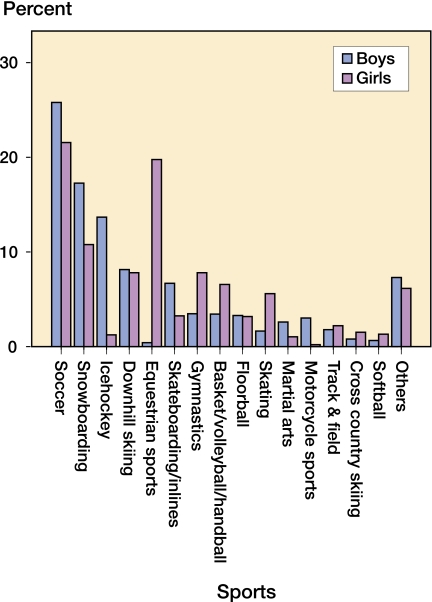
Sports-related injuries in each sex, broken down by the type of sport practiced at injury.

The most common sport associated with fractures in both sexes was soccer. Soccer-related fractures increased in the last decade from 18 per 10^4^ in 1998 to 29 per 10^4^ in 2007, with an incidence ratio of 1.6 (CI: 1.1–2.0). Fractures related to equestrian sports mainly occurred in girls, and those related to ice hockey mainly occurred in boys. The distribution of sports-related injuries for each sex is shown in Figure 4.

## Discussion

We found a 59% increase in incidence over time, which is remarkable and requires scrutiny. The database was cross-checked with the hospital's inpatient registry, so the percentage of fractures resulting in hospitalization that were lost to registration should have been small and near constant over time. The lack of registration for all fractures, not only those admitted, was evaluated between 1998 and 2007. Thus, comparisons over time are more reliable between these years. Between 1998 and 2007, the incidence of all fractures increased from 213 to 240, with an incidence ratio of 1.1 (CI: 1.0–1.3). The incidence of fractures requiring admission increased by 38% during the same period (from 37 to 51), with an incidence ratio of 1.4 (CI: 1.1–1.8). The greater increase in fractures requiring admission could indicate more serious injuries. It could also be that the indications for admission were more liberal in the later part of the period. However, the average length of stay in hospital was similar over time. Also, the percentage of fractures caused by slight trauma but leading to admission decreased while the percentage of fractures caused by moderate and severe trauma increased. We therefore believe that there was a real increase in fracture incidence between 1998 and 2007.

Why did this increase occur? A recent study by Raustorp and Ludvigsson (2007) showed an increase in daily physical activity in Swedish school children between 2000 and 2006. Also, they found that physical activity levels were not related to BMI. They concluded that the change in activity levels was due to an increased awareness of the importance of physical activity in daily life, and implementation of programs to enhance such activities. The Swedish Sports Council reported a 5% increase in participation in organized sports among teenagers when comparing the years 1998 and 2005, and that children become involved in organized sporting activities at a younger age (Swedish Sports Council 2005). Perhaps Swedish children are more active today than in the 1990's when Tiderius and colleagues (1999) reasoned that the decrease in fracture incidence seen in Malmö between the 1970's and the 1990's could be partly explained by a reduced level of spontaneous activity. They referred to Engström (1996) who had reported a reduction in spontaneous playing activities in Swedish children.

Petersen and colleagues (2003) showed an increased prevalence of overweight (age-specific BMI corresponding to BMI > 25 at age 18) among children in Umeå between 1986 and 2001. Overweight children are reported to be at increased risk of sustaning fractures (Goulding et al. 1998, Taylor et al. 2006). Hypothetical explanations for this are greater mechanical loads when falling, low bone strength relative to body mass (Wetzsten et al. 2008) and poor balance (Goulding et al. 2002). Could it be that the increased incidence of fractures is partly explained by increased activity levels among some children and increased prevalence of overweight among others?

When comparing incidence with that in previous studies, one must bear in mind that the most recent studies have looked at individuals from 0 to 16 years of age (Table 1). It can be argued that children are in most cases skeletally mature by the age of 16. However, the incidence of fractures in the late teenage years is still high, and is not reached again until the 50s in women and the late 70s in men (Bergström et al. 2008). For comparative reasons, we calculated the overall incidence for the population aged 0–16 years and the accumulated risk until 16 years of age.

In comparing our results with those of Landin (1983) and Tiderius et al. (1999), some differences were to be expected given the differences in climate and demographics. However, the incidence figures were remarkably similar. Lyons et al. (2000) reported differences in incidence between Scandinavian populations and a Welsh population (Table 1). Cooper et al. (2004) reported an incidence ratio of 1.7 between districts in Northern Ireland and districts in south-eastern England.

The general predominance of boys is in line with previous results. The reason for this predominance is probably a combination of biological factors and social, gender-related differences related to activity and risk taking. Identification of these factors was not possible in this study, but it could be of value in the future, e.g. to help identify fracture-prone individuals in both sexes, and to aid in targeting of preventive measures.

The difference in timing of the peak incidence for boys and girls reflects differences in growth between the sexes. The peak incidence coincides with the pubertal growth spurt, when there is a relative decrease in bone mineral density due to bone expansion and insufficient mineralization (Faulkner et al. 2006). The age for the pubertal peak height velocity in the Swedish population is 14 years in boys and 12 years in girls (Albertsson Wikland et al. 2002).

Seasonal variations for all fractures have been described by other authors (Landin 1983, Tiderius et al. 1999, Lyons et al. 2000). A unique feature of the present data is the high incidence of fractures in March. This is due to “sports vacations”, a week at the beginning of March when children are traditionally encouraged to take part in winter sports. We found that December is the month with the lowest number of fractures and also one of the months with the lowest percentage of outdoor fractures (47%), which can be explained by the lack of daylight in December. The percentage of outdoor fractures in June, on the other hand, when the days are long and outdoor activities are more frequent was 81%.

As in previous articles (Table 1), we found that the most common fracture site was the distal forearm.

During the first years of life, children undergo dramatic developments in motor skills. In their explorations, they tend to fall off furniture and staircases in the first year of life, whereas pre-adolescence children fall off playground equipment, bikes, and other structures. This is reflected in the distribution of injury mechanisms.

The percentage of fractures caused by severe or slight trauma was similar to the corresponding results of Landin (1983) and Tiderius et al. (1999). We categorized a greater proportion of events as being caused by moderate trauma (falls from 0.5–3 m, e.g. falls from bicycles, horses, bunk-beds, or swings). This was perhaps because we could categorize fewer events as “unknown”.

Children start to participate in organized sports between the ages of 5 and 7. This was reflected in the distribution of sports-related fractures, which increased after the age of 5. Sports accounted for more fractures in our material than in the studies by Landin (1997), Tiderius et al. (1999), and Rennie et al. (2007) but there was a similar percentage of fractures to that reported by Lyons et al. (1999) in a Welsh population. This Welsh population showed a higher fracture incidence than Scandinavian populations for the same year, and the authors discussed whether this could be explained by a higher participation in sports. They concluded that it could not be taken as the sole explanation. Not even a twofold increase in sports and leisure activities could explain the higher incidence. Our findings of a higher percentage of sports-related fractures compared to those of Landin and Tiderius et al. are most likely a reflection of differences in registration of data, but they are perhaps also partly due to an increase in participation in organized sporting activities. Comparisons of activity at injury with previous studies are difficult because of differences in the manner of categorization.

Figures from the Swedish Sports Council show that the most common sports practiced in organized forms by girls are soccer, equestrian sports, and floorball. For boys, they are soccer, floorball, and ice hockey (Swedish Sports Council 2005). As expected, soccer accounted for the greatest number of fractures in both sexes. Soccer-related injuries increased over a 10-year period, in both boys and girls, most likely due to an increased participation in organized soccer.

The differences between sexes in the distribution of fractures related to equestrian sports and ice hockey were in accordance with the sex-related distribution of participants. We were unable to identify any organized sport that was associated with a disproportionally high number of fracture events.

The percentage of traffic-related injuries was similar to that in previous Swedish reports (Landin 1983, Tiderius et al. 1999).

One limitation of the study is that it involved one center only, so our interpretation of the results only applies to this study population.

This study shows that fractures are common in children in Umeå, with one-third of all children sustaining at least one fracture before the age of 17 years. There was an increase in incidence among children and adolescents between 1998 and 2007. We were unable to identify a single activity or mechanism of injury responsible for this increase, and unfortunately this makes it difficult to suggest implementation of any specific preventive measures. Mechanisms and activities at injury vary with age; this reflects the development of social and motor skills and the changing activities of children as they grow older. The fracture pattern is also affected by changes in the mechanical properties of bone during growth, length of day, climate, trends in leisure activities, and even local holidays.
